# A Computing Method to Determine the Performance of an Ionic Liquid Gel Soft Actuator

**DOI:** 10.1155/2018/8327867

**Published:** 2018-05-02

**Authors:** Bin He, Chenghong Zhang, Yanmin Zhou, Zhipeng Wang

**Affiliations:** College of Electronics and Information Engineering, Tongji University, No. 4800 Caoan Road, Shanghai 201804, China

## Abstract

A new type of soft actuator material—an ionic liquid gel (ILG) that consists of BMIMBF_4_, HEMA, DEAP, and ZrO_2_—is polymerized into a gel state under ultraviolet (UV) light irradiation. In this paper, we first propose that the ILG conforms to the assumptions of hyperelastic theory and that the Mooney-Rivlin model can be used to study the properties of the ILG. Under the five-parameter and nine-parameter Mooney-Rivlin models, the formulas for the calculation of the uniaxial tensile stress, plane uniform tensile stress, and 3D directional stress are deduced. The five-parameter and nine-parameter Mooney-Rivlin models of the ILG with a ZrO_2_ content of 3 wt% were obtained by uniaxial tensile testing, and the parameters are denoted as *c*_10_, *c*_01_, *c*_20_, *c*_11_, and *c*_02_ and *c*_10_, *c*_01_, *c*_20_, *c*_11_, *c*_02_, *c*_30_, *c*_21_, *c*_12_, and *c*_03_, respectively. Through the analysis and comparison of the uniaxial tensile stress between the calculated and experimental data, the error between the stress data calculated from the five-parameter Mooney-Rivlin model and the experimental data is less than 0.51%, and the error between the stress data calculated from the nine-parameter Mooney-Rivlin model and the experimental data is no more than 8.87%. Hence, our work presents a feasible and credible formula for the calculation of the stress of the ILG. This work opens a new path to assess the performance of a soft actuator composed of an ILG and will contribute to the optimized design of soft robots.

## 1. Introduction

At room temperature, the ionogel is a polymerized gelatinous mixture from the ionic liquid and polymer matrix under UV irradiation [1, 2]. The high conductivity and stability of ionic liquids and the good mechanical properties of polymers make ionic liquid gels an ideal replacement for the traditional electroactive polymers [3]. Due to their high environmental adaptability and low-pressure impedance characteristics, soft robots have shown broad application potential in the fields of biology, medicine, agriculture, and so on. The adoption of electroactive polymer (EAP) materials for soft robots has become a hot research topic in recent years. Progress in electrochemical actuators has been made over the past few decades due to their desirable mechanical properties for intelligent robots, which are an alternative for air- and fluid-derived equipment [4–7]. Ionic liquid gels (ILG) are suitable building blocks for advanced actuators due to their tunable ionic conductivity, chemical stability, thermal stability, and simple ion transport [8-9].

Electrochemical actuators have been further developed over the past few decades for their desirable mechanical properties in intelligent robots, which are alternatives to air- and fluid-derived devices. The flexible ionic conductivity of ILG is better suited to the evolution of building blocks due to the simpler ion transport actuators [10].

Noncovalent interactions provide the gels with the very high mechanical strength and excellent self-healing ability of supramolecular materials [11, 12]. Based on these studies, we used ZrO_2_ to fabricate supramolecular nanocomposites with the electrochemical behavior of an ionic liquid and mechanical strength of an ionogel polymer.

Among numerical approaches, finite element analysis is one of the most effective and most common information extraction methods to evaluate and optimize robot designs. By using this method, analytical models can be greatly simplified, which greatly increases the computational efficiency. The largest drawback is that ignorance of the nonlinear and constitutive model and the simplification of the computational model often lead to coarse solutions [13, 14]. Numerical simulations offer sufficient insight for each case during general soft robot design.

Lee et al. used the finite element method (FEM) to successfully predict the mechanical behavior of an ionic polymer-metal composite (IPMC) actuator [15]. Wang et al. established a model based on the FEM to determine the electromechanical bending behavior of photocurable ionogel actuators (PIA) [16]. He et al. used the common large-scale finite element analysis software ANSYS to simulate an ILG, which is based on the SOLID186 element and the nonlinear hyperelastic Mooney-Rivlin model [17].

Because the use of an ILG actuator requires deep understanding of the mechanical properties of the soft robot, it is necessary to establish a theoretical model to obtain its performance index. To reduce the experimental cost and time, we systematically analyzed the ILG via numerical simulations and verified the accuracy of the calculation, which contributed to the development of the ILG in the soft robot. The numerical simulation results matched the corresponding experiments, proving the validity of the model [18, 19].

## 2. Fabrication of the Ionic Liquid Gel

In the experiments, the ILG was composed of 1-butyl-3-methylimidazolium tetrafluoroborate (BMIMBF_4_), hydroxyethyl methacrylate (HEMA), 2-diethoxyacetophenone (DEAP), and ZrO_2_, with masses of 900 mg, 68.6 mg, 1.4 mg, and 30 mg, respectively. The mixed solution was then placed into a magnetic stirrer to form a suspension. Following that, the sample was placed on an ML-3500C Maxima-type cold light source for ultra-high-intensity UV radiation curing to induce polymerization. The UV intensity is 90000uW / cm^2^ (15^*″*^/ 380 mm distance) [20].

**Figure 1:**
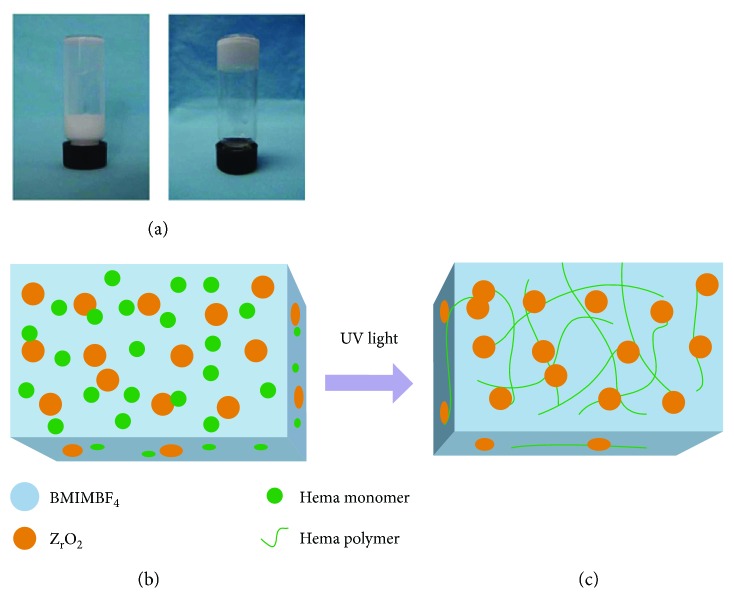
The proposed mechanism of the BMIMBF_4_-based ionogel under ultraviolet light. (a) ILG solution and ILG solid, respectively. (b) The ILG solution includes BMIMBF_4_, HEMA, DEAP, and ZrO_2_. (c) HEMA and ZrO_2_ are cross-coupled to form a 3D network.

Figure 1(a) shows a comparison of the morphology of the ionic liquid before and after gel formation: the left figure shows the liquid state, while the right shows the solid state formed after polymerization. The schematic diagram of the principle behind gel formation is illustrated in Figures 1(b) and 1(c). Under UV light irradiation and the action of the DEAP catalyst, the polymer matrix was cross-linked into a porous network structure.

## 3. Material Nonlinearity and Parameters

**Figure 2:**
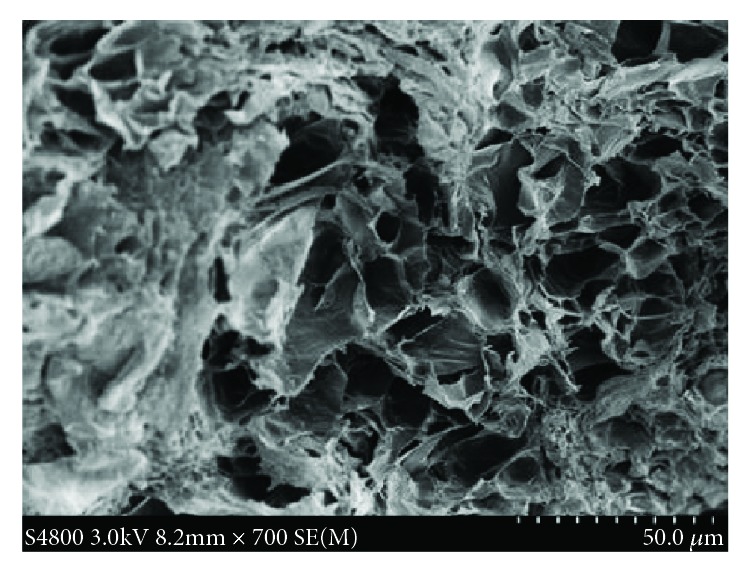
SEM image of a freeze-dried BMIMBF_4_-based gel after replacing the ionic liquid with water [17].

The morphological analysis of the freeze-dried sample by scanning electron microscopy (SEM) showed that a porous microstructure was present throughout the ionogel. Distilled water was used to replace the internal ionic liquid in the ILG after freeze-drying treatment, and then, an S4800 Hitachi high-resolution field-emission scanning electron microscope was used to scan the sample.  Figure 2 shows the spatial structure of the ionic liquid carrier HEMA for the typical 3D porous structure with a 5000x magnification, in which its matrix is cross-linked to generate a 3D support skeleton, offering good mechanical strength and self-repairing performance. Due to the porous 3D network in the ionogel, gelated BMIMBF_4_ retains a relatively high ionic conductivity.

### 3.1. Hyperelastic Hypothesis

Assuming that the ILG is an isotropic, incompressible, hyperelastic body, the following assumptions can be made based on the theory of continuum mechanics to study its mechanical properties.

1. The strain energy function *W* of a unit mass of material is an analytic function of the strain tensor of the natural state, termed the hyperelastic hypothesis. If the rate of change of *W* is equal to the power of the stress, then the material is a hyperelastic material. The mechanical properties of a hyperelastic material are described by the strain energy density function *W*, which has many functions.
2. Isotropy can be assumed.
3. The volume of the material before and after deformation can be assumed to be the same.

*λ*_1_, *λ*_2_, and *λ*_3_ are set as the *x*, *y*, and *z* directions of the main (extension) deformation rate, respectively, given by

(1) $$\begin{array}{c} \lambda_{1}=\frac{x}{x_{0}},\\ \lambda_{2}=\frac{y}{y_{0}},\\ \lambda_{3}=\frac{d}{d_{0}}, \end{array}$$

where *x*, *y*, and *d* are the length, width, and thickness, respectively, and *x*_0_, *y*_0_, and *d*_0_ are the corresponding initial values before deformation.

Because the material is incompressible, its volume is the same before and after deformation, giving

(2) $$\begin{array}{c} xyd=x_{0}y_{0}d_{0}, \end{array}$$

namely,

(3) $$\begin{array}{c} \frac{xyd}{x_{0}y_{0}d_{0}}=\lambda_{1}\lambda_{2}\lambda_{3}=1. \end{array}$$

### 3.2. Hyperelastic Stress

**Figure 3:**
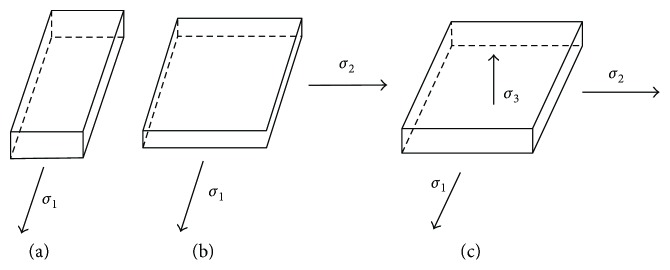
Analyses of the stress in different states.

Considering the mechanical performance requirements for potential applications, three kinds of deformation states should be examined: Figure 3(a) shows the uniaxial tensile stress, Figure 3(b) shows uniform prestretching in the *X* and *Y* directions, and Figure 3(c) shows the Maxwell stress increased along the thickness.

The physical properties are mainly expressed by the strain energy function, and each model is a special form of this function [21–23]. Once the form of the strain energy function *W* is determined, the Cauchy stress tensor **P** can be given by

(4) $$\begin{array}{c} \sigma =-pI+2\frac{\partial W}{\partial I_{1}}B-2\frac{\partial W}{\partial I_{2}}B^{-1}, \end{array}$$

where **I** is the unit tensor, which is the left Gauss deformation tensor, and *p* is the hydrostatic pressure resulting from the assumption of incompressibility.

(5) $$\begin{array}{c} I_{1}=B,\\ I_{2}=\frac{1}{2}\left[I_{1}^{2}-t_{r}\left(B^{2}\right)\right],\\ I_{3}=\det B, \end{array}$$

where *B* is the component of the Green strain tensor. The relationship between the invariants and the principal elongation is of a function of *B*.

(6) $$\begin{array}{c} I_{1}=t_{r}\left[B\right]=B_{ii}=\lambda_{1}^{2}+\lambda_{2}^{2}+\lambda_{3}^{2},\\ I_{2}=\frac{1}{2}{\left(t_{r}\left[B\right]\right)}^{2}-{\left(t_{r}\left[B\right]\right)}^{2}=\frac{1}{2}\left(B_{ii}B_{ii}-B_{ij}B_{ji}\right)=\lambda_{1}^{2}\lambda_{2}^{2}+\lambda_{2}^{2}\lambda_{3}^{2}+\lambda_{3}^{2}\lambda_{1}^{2}=\frac{1}{\lambda_{1}^{2}}+\frac{1}{\lambda_{2}^{2}}+\frac{1}{\lambda_{3}^{2}},\\ I_{3}=\det B=\lambda_{1}^{2}\lambda_{2}^{2}\lambda_{3}^{2}. \end{array}$$

Hence, the isotropic and incompressible deformation process of an ILG is given as

(7) $$\begin{array}{c} \sqrt{I_{3}}=\lambda_{1}\lambda_{2}\lambda_{3}=1. \end{array}$$

According to (4) and (6), we can obtain

(8) $$\begin{array}{c} \sigma_{i}=2\lambda_{i}^{2}\left[\frac{\partial W}{\partial I_{1}}-\frac{1}{\lambda_{i}^{2}}\frac{\partial W}{\partial I_{2}}\right]-p, \end{array}$$

where *I*_1_, *I*_2_, and *I*_3_ are the relative changes in the length, surface area, and volume of the elastomer, respectively.

### 3.3. Mooney-Rivlin Model

The Mooney-Rivlin model was chosen after comparing various hyperelastic constitutive models. The mechanical properties of ionic gel materials can be studied by using the Mooney-Rivlin formula, which is considered to be a nonlinear finite element of ionic gels in this study [24–26].

The strain energy function in the Mooney-Rivlin model equation is as follows:

(9) $$\begin{array}{c} W=\overset{n}{\underset{i\neq j}{\sum }}c_{ij}{\left(I_{1}-3\right)}^{i}{\left(I_{2}-3\right)}^{j}, \end{array}$$

where *c*_*ij*_ is a constant.

The Mooney-Rivlin model is the most widely used strain energy function in the finite element method. It assumes that the strain energy density is a first-order function of the principal strain constant. Under large deformations, the mechanical properties of the ILG, as an incompressible hyperelastic material, are described.

### 3.4. Selection of the Constitutive Model for the ILG

Yeoh noted that the practical value of the higher-order strain energy function is small because the reproducibility of the ILG material is not sufficient and does not allow accurate estimation of a large number of parameters [27].

Due to its simplicity and practicality, the Mooney-Rivlin model is widely used in finite element analysis. The first-order Mooney-Rivlin model describes the mechanical properties of incompressible hyperelastic materials under large deformation. The higher-order Mooney-Rivlin model can obtain a good approximation for the solution of large strain.

The mechanical response of the hyperelastic material model is determined by the strain energy density function. The Mooney-Rivlin constant of the material must be accurately evaluated to obtain a reliable result from the hyperelastic analysis. In finite element analysis, the hyperelastic material is generally assumed to be a homogeneous isotropic material whose elastic modulus (Young's modulus) *E*, initial shear modulus *G*_0_, and Poisson's ratio *v* satisfy the following relationship [28, 29].

(10) $$\begin{array}{c} E=2G_{0}\left(1+v\right). \end{array}$$

## 4. Stress Calculation

### 4.1. Uniaxial Tension (State I)

#### 4.1.1. Five-Parameter Mooney-Rivlin Model

From (9), the strain energy equation in the five-parameter Mooney-Rivlin model can be written as

(11) $$\begin{array}{c} W=c_{10}\left(I_{1}-3\right)+c_{01}\left(I_{2}-3\right)+c_{20}{\left(I_{1}-3\right)}^{2}+c_{11}\left(I_{1}-3\right)\left(I_{2}-3\right)+c_{02}{\left(I_{2}-3\right)}^{2}. \end{array}$$

From (8), we get

(12) $$\begin{array}{c} \sigma_{i}=2\left(\lambda_{i}^{2}\frac{\partial W}{\partial I_{1}}-\frac{1}{\lambda_{i}^{2}}\frac{\partial W}{\partial I_{2}}\right)-p. \end{array}$$

The stresses in all directions are

(13) $$\begin{array}{c} \sigma_{1}=2\left(\lambda_{1}^{2}\frac{\partial W}{\partial I_{1}}-\frac{1}{\lambda_{1}^{2}}\frac{\partial W}{\partial I_{2}}\right)-p, \end{array}$$

(14) $$\begin{array}{c} \sigma_{2}=2\left(\lambda_{2}^{2}\frac{\partial W}{\partial I_{1}}-\frac{1}{\lambda_{2}^{2}}\frac{\partial W}{\partial I_{2}}\right)-p, \end{array}$$

(15) $$\begin{array}{c} \sigma_{3}=2\left(\lambda_{3}^{2}\frac{\partial W}{\partial I_{1}}-\frac{1}{\lambda_{3}^{2}}\frac{\partial W}{\partial I_{2}}\right)-p, \end{array}$$

where *∂W*/*∂I*_1_ and *∂W*/*∂I*_2_ are the partial differentials in the strain energy function *W* for *I*_1_ and *I*_2_, respectively, and *σ*_1_, *σ*_2_, and *σ*_3_ are the stresses in the *x*, *y*, and *z* directions, respectively.

When uniformly stretched in the *X* direction, *λ*_2_ = *λ*_3_, and from (3), we get

(16) $$\begin{array}{c} \lambda_{2}=\lambda_{3}=\frac{1}{\sqrt{\lambda_{1}}}. \end{array}$$

From (6) and (16), we obtain

(17) $$\begin{array}{c} I_{1}=\lambda_{1}^{2}+\frac{2}{\lambda_{1}},\\ I_{2}=\frac{1}{\lambda_{1}^{2}}+2\lambda_{1}. \end{array}$$

Because only axial tensile deformation is considered, the stress in the other two directions is zero.

(18) $$\begin{array}{c} \sigma_{2}=\sigma_{3}=0. \end{array}$$

From (14) or (15) and (16), we obtain

(19) $$\begin{array}{c} p=2\left(\frac{1}{\lambda_{1}}\frac{\partial W}{\partial I_{1}}-\lambda_{1}\frac{\partial W}{\partial I_{2}}\right). \end{array}$$

Substituting (19) into (13),

(20) $$\begin{array}{c} \sigma_{1}=2\left(\lambda_{1}^{2}-\frac{1}{\lambda_{1}}\right)\left(\frac{\partial W}{\partial I_{1}}+\frac{1}{\lambda_{1}}\frac{\partial W}{\partial I_{2}}\right). \end{array}$$

For (11), the partial differentials of the strain energy function *W* for *I*_1_ and *I*_2_ are given by

(21) $$\begin{array}{c} \frac{\partial W}{\partial I_{1}}=c_{10}+2c_{02}\left(I_{1}-3\right)+c_{11}\left(I_{2}-3\right),\\ \frac{\partial W}{\partial I_{2}}=c_{01}+2c_{02}\left(I_{2}-3\right)+c_{11}\left(I_{1}-3\right). \end{array}$$

Therefore,

(22) $$\begin{array}{c} \sigma_{1}=2\left(\lambda_{1}^{2}-\frac{1}{\lambda_{1}}\right)\left\{c_{10}+2c_{02}\left(I_{1}-3\right)+c_{11}\left(I_{2}-3\right)+\frac{1}{\lambda_{1}}\left[c_{01}+2c_{02}\left(I_{2}-3\right)+c_{11}\left(I_{1}-3\right)\right]\right\}. \end{array}$$

#### 4.1.2. Nine-Parameter Mooney-Rivlin Model

From (9), the strain energy equation in the nine-parameter Mooney-Rivlin model is

(23) $$\begin{array}{c} W=c_{10}\left(I_{1}-3\right)+c_{01}\left(I_{2}-3\right)+c_{20}{\left(I_{1}-3\right)}^{2}+c_{11}\left(I_{1}-3\right)\left(I_{2}-3\right)+c_{02}{\left(I_{2}-3\right)}^{2}+c_{30}{\left(I_{1}-3\right)}^{3}+c_{21}{\left(I_{1}-3\right)}^{2}\left(I_{2}-3\right)+c_{12}\left(I_{1}-3\right){\left(I_{2}-3\right)}^{2}+c_{03}{\left(I_{2}-3\right)}^{3}. \end{array}$$

For (23), the partial differentials of the strain energy function *W* for *I*_1_ and *I*_2_ are given by

(24) $$\begin{array}{c} \frac{\partial W}{\partial I_{1}}=c_{10}+2c_{20}\left(I_{1}-3\right)+c_{11}\left(I_{2}-3\right)+3c_{30}{\left(I_{1}-3\right)}^{2}+2c_{21}\left(I_{1}-3\right)\left(I_{2}-3\right)+c_{12}{\left(I_{2}-3\right)}^{2}=M,\\ \frac{\partial W}{\partial I_{2}}=c_{01}+2c_{02}\left(I_{2}-3\right)+c_{11}\left(I_{1}-3\right)+3c_{03}{\left(I_{2}-3\right)}^{2}+2c_{12}\left(I_{1}-3\right)\left(I_{2}-3\right)+c_{21}{\left(I_{1}-3\right)}^{2}=N. \end{array}$$

Therefore,

(25) $$\begin{array}{c} \sigma_{1}=2\left(\lambda_{1}^{2}-\frac{1}{\lambda_{1}}\right)\left(M+\frac{1}{\lambda_{1}}N\right). \end{array}$$

### 4.2. Evenly Stretched in the *X* and *Y* Directions (State II)

#### 4.2.1. Five-Parameter Mooney-Rivlin Model

From (9), the strain energy equation in the five-parameter Mooney-Rivlin model is

(26) $$\begin{array}{c} W=c_{10}\left(I_{1}-3\right)+c_{01}\left(I_{2}-3\right)+c_{20}{\left(I_{1}-3\right)}^{2}+c_{11}\left(I_{1}-3\right)\left(I_{2}-3\right)+c_{02}{\left(I_{2}-3\right)}^{2}. \end{array}$$

From (8), we get

(27) $$\begin{array}{c} \sigma_{i}=2\left(\lambda_{i}^{2}\frac{\partial W}{\partial I_{1}}-\frac{1}{\lambda_{i}^{2}}\frac{\partial W}{\partial I_{2}}\right)-p. \end{array}$$

The stresses in all directions are

(28) $$\begin{array}{c} \sigma_{1}=2\left(\lambda_{1}^{2}\frac{\partial W}{\partial I_{1}}-\frac{1}{\lambda_{1}^{2}}\frac{\partial W}{\partial I_{2}}\right)-p, \end{array}$$

(29) $$\begin{array}{c} \sigma_{2}=2\left(\lambda_{2}^{2}\frac{\partial W}{\partial I_{1}}-\frac{1}{\lambda_{2}^{2}}\frac{\partial W}{\partial I_{2}}\right)-p, \end{array}$$

(30) $$\begin{array}{c} \sigma_{3}=2\left(\lambda_{3}^{2}\frac{\partial W}{\partial I_{1}}-\frac{1}{\lambda_{3}^{2}}\frac{\partial W}{\partial I_{2}}\right)-p. \end{array}$$

When uniformly stretched in the *X* and *Y* directions, *λ*_1_ = *λ*_2_, and from (3), we obtain

(31) $$\begin{array}{c} \lambda_{1}=\lambda_{2}=\frac{1}{\sqrt{\lambda_{3}}}. \end{array}$$

From (6) and (31), we get

(32) $$\begin{array}{c} I_{1}=2\lambda_{1}^{2}+\frac{2}{\lambda_{1}^{4}},\\ I_{2}=\frac{2}{\lambda_{1}^{2}}+\lambda_{1}^{4}. \end{array}$$

Because tensile deformations are only considered in the *X* and *Y* directions, the stress in the thickness direction is zero.

(33) $$\begin{array}{c} \sigma_{1}=\sigma_{2},\\ \sigma_{3}=0. \end{array}$$

From (28) or (29) and (31), we obtain

(34) $$\begin{array}{c} p=2\left(\frac{1}{\lambda_{1}^{4}}\frac{\partial W}{\partial I_{1}}-\lambda_{1}^{4}\frac{\partial W}{\partial I_{2}}\right). \end{array}$$

Substituting (34) into (28) or (29),

(35) $$\begin{array}{c} \sigma_{1}=\sigma_{2}=2\left(\lambda_{1}^{2}-\frac{1}{\lambda_{1}^{4}}\right)\left(\frac{\partial W}{\partial I_{1}}+\lambda_{1}^{2}\frac{\partial W}{\partial I_{2}}\right). \end{array}$$

For (26), the partial differentials of the strain energy function *W* for *I*_1_ and *I*_2_ are given by

(36) $$\begin{array}{c} \frac{\partial W}{\partial I_{1}}=c_{10}+2c_{20}\left(I_{1}-3\right)+c_{11}\left(I_{2}-3\right),\\ \frac{\partial W}{\partial I_{2}}=c_{01}+2c_{02}\left(I_{2}-3\right)+c_{11}\left(I_{1}-3\right). \end{array}$$

Therefore,

(37) $$\begin{array}{c} \sigma_{1}=\sigma_{2}=2\left(\lambda_{1}^{2}-\frac{1}{\lambda_{1}^{4}}\right)\left\{c_{10}+2c_{02}\left(I_{1}-3\right)+c_{11}\left(I_{2}-3\right)+\lambda_{1}^{2}\left[c_{01}+2c_{02}\left(I_{2}-3\right)+c_{11}\left(I_{1}-3\right)\right]\right\}. \end{array}$$

#### 4.2.2. Nine-Parameter Mooney-Rivlin Model

From (9), the strain energy equation of the nine-parameter Mooney-Rivlin model is

(38) $$\begin{array}{c} W=c_{10}\left(I_{1}-3\right)+c_{01}\left(I_{2}-3\right)+c_{20}{\left(I_{1}-3\right)}^{2}+c_{11}\left(I_{1}-3\right)\left(I_{2}-3\right)+c_{02}{\left(I_{2}-3\right)}^{2}+c_{30}{\left(I_{1}-3\right)}^{3}+c_{21}{\left(I_{1}-3\right)}^{2}\left(I_{2}-3\right)+c_{12}\left(I_{1}-3\right){\left(I_{2}-3\right)}^{2}+c_{03}{\left(I_{2}-3\right)}^{3}. \end{array}$$

For (38), the partial differentials of the strain energy function *W* for *I*_1_ and *I*_2_ are given by

(39) $$\begin{array}{c} \frac{\partial W}{\partial I_{1}}=c_{10}+2c_{20}\left(I_{1}-3\right)+c_{11}\left(I_{2}-3\right)+3c_{30}{\left(I_{1}-3\right)}^{2}+2c_{21}\left(I_{1}-3\right)\left(I_{2}-3\right)+c_{12}{\left(I_{2}-3\right)}^{2}=M.\\ \frac{\partial W}{\partial I_{2}}=c_{01}+2c_{02}\left(I_{2}-3\right)+c_{11}\left(I_{1}-3\right)+3c_{03}{\left(I_{2}-3\right)}^{2}+2c_{12}\left(I_{1}-3\right)\left(I_{2}-3\right)+c_{21}{\left(I_{1}-3\right)}^{2}=N. \end{array}$$

Therefore,

(40) $$\begin{array}{c} \sigma_{1}=\sigma_{2}=2\left(\lambda_{1}^{2}-\frac{1}{\lambda_{1}^{4}}\right)\left(M+\lambda_{1}^{2}N\right). \end{array}$$

### 4.3. Applied Maxwell Stress Loading (State III)

The Maxwell stress can be written as

(41) $$\begin{array}{c} p=-{\varsigma \varsigma }_{0}V^{2}=-{\varsigma \varsigma }_{0}\frac{u^{2}}{d^{2}}, \end{array}$$

where *ς* is the insulation constant, *ς*_0_ is the vacuum dielectric constant (8.85 × 10^−12^F/m), *V* is the electric field intensity, and *u* is the voltage.

#### 4.3.1. Five-Parameter Mooney-Rivlin Model

From (9), the strain energy equation in the five-parameter Mooney-Rivlin model is

(42) $$\begin{array}{c} W=c_{10}\left(I_{1}-3\right)+c_{01}\left(I_{2}-3\right)+c_{20}{\left(I_{1}-3\right)}^{2}+c_{11}\left(I_{1}-3\right)\left(I_{2}-3\right)+c_{02}{\left(I_{2}-3\right)}^{2}. \end{array}$$

From (8), we get

(43) $$\begin{array}{c} \sigma_{i}=2\left(\lambda_{i}^{2}\frac{\partial W}{\partial I_{1}}-\frac{1}{\lambda_{i}^{2}}\frac{\partial W}{\partial I_{2}}\right)-p. \end{array}$$

The stresses in all directions are

(44) $$\begin{array}{c} \sigma_{1}=2\left(\lambda_{1}^{2}\frac{\partial W}{\partial I_{1}}-\frac{1}{\lambda_{1}^{2}}\frac{\partial W}{\partial I_{2}}\right)-p, \end{array}$$

(45) $$\begin{array}{c} \sigma_{2}=2\left(\lambda_{2}^{2}\frac{\partial W}{\partial I_{1}}-\frac{1}{\lambda_{2}^{2}}\frac{\partial W}{\partial I_{2}}\right)-p, \end{array}$$

(46) $$\begin{array}{c} \sigma_{3}=2\left(\lambda_{3}^{2}\frac{\partial W}{\partial I_{1}}-\frac{1}{\lambda_{3}^{2}}\frac{\partial W}{\partial I_{2}}\right)-p. \end{array}$$

When the Maxwell stress is loaded, namely, *λ*_1_ = *λ*_2_, (3) gives

(47) $$\begin{array}{c} \lambda_{1}=\lambda_{2}=\frac{1}{\sqrt{\lambda_{3}}}. \end{array}$$

From (6) and (45), we get

(48) $$\begin{array}{c} I_{1}=2\lambda_{1}^{2}+\frac{2}{\lambda_{1}^{4}},\\ I_{2}=\frac{2}{\lambda_{1}^{2}}+\lambda_{1}^{4}. \end{array}$$

For (42), the partial differentials of the strain energy function *W* for *I*_1_ and *I*_2_ are given by

(49) $$\begin{array}{c} \frac{\partial W}{\partial I_{1}}=c_{10}+2c_{20}\left(I_{1}-3\right)+c_{11}\left(I_{2}-3\right),\\ \frac{\partial W}{\partial I_{2}}=c_{01}+2c_{02}\left(I_{2}-3\right)+c_{11}\left(I_{1}-3\right). \end{array}$$

Because the materials uniformly stretched in the *X* and *Y* directions,

(50) $$\begin{array}{c} \sigma_{1}=\sigma_{2}. \end{array}$$

From (41), we obtain

(51) $$\begin{array}{c} \sigma_{3}=-{\varsigma \varsigma }_{0}V^{2}=-{\varsigma \varsigma }_{0}\frac{u^{2}}{d^{2}}=-{\varsigma \varsigma }_{0}\frac{u^{2}}{{\left(\lambda_{3}d_{0}\right)}^{2}}. \end{array}$$

Substituting (49), (50), and (51) into (44) and (46),

(52) $$\begin{array}{c} \sigma_{1}=\sigma_{2}=2\left\{\lambda_{1}^{2}\left[c_{10}+2c_{20}\left(I_{1}-3\right)+c_{11}\left(I_{2}-3\right)\right]-\frac{1}{\lambda_{1}^{2}}\left[c_{01}+2c_{02}\left(I_{2}-3\right)+c_{11}\left(I_{1}-3\right)\right]\right\}-p, \end{array}$$

(53) $$\begin{array}{c} \sigma_{3}=2\left\{\frac{1}{\lambda_{1}^{4}}\left[c_{10}+2c_{20}\left(I_{1}-3\right)+c_{11}\left(I_{2}-3\right)\right]-\lambda_{1}^{4}\left[c_{01}+2c_{02}\left(I_{2}-3\right)+c_{11}\left(I_{1}-3\right)\right]\right\}-p, \end{array}$$

(54) $$\begin{array}{c} \sigma_{3}=-{\varsigma \varsigma }_{0}\frac{u^{2}}{{\left(\lambda_{3}d_{0}\right)}^{2}}=-{\varsigma \varsigma }_{0}\frac{\lambda_{1}^{4}u^{2}}{{d_{0}}^{2}}. \end{array}$$

From (53) and (54),

(55) $$\begin{array}{c} p=2\left\{\frac{1}{\lambda_{1}^{4}}\left[c_{10}+2c_{20}\left(I_{1}-3\right)+c_{11}\left(I_{2}-3\right)\right]-\lambda_{1}^{4}\left[c_{01}+2c_{02}\left(I_{2}-3\right)+c_{11}\left(I_{1}-3\right)\right]\right\}+{\varsigma \varsigma }_{0}\frac{\lambda_{1}^{4}u^{2}}{{d_{0}}^{2}}. \end{array}$$

Therefore,

(56) $$\begin{array}{c} \sigma_{1}=\sigma_{2}=2\left\{\lambda_{1}^{2}\left[c_{10}+2c_{20}\left(I_{1}-3\right)+c_{11}\left(I_{2}-3\right)\right]-\frac{1}{\lambda_{1}^{2}}\left[c_{01}+2c_{02}\left(I_{2}-3\right)+c_{11}\left(I_{1}-3\right)\right]\right\}-2\left\{\frac{1}{\lambda_{1}^{4}}\left[c_{10}+2c_{20}\left(I_{1}-3\right)+c_{11}\left(I_{2}-3\right)\right]-\lambda_{1}^{4}\left[c_{01}+2c_{02}\left(I_{2}-3\right)+c_{11}\left(I_{1}-3\right)\right]\right\}-{\varsigma \varsigma }_{0}\frac{\lambda_{1}^{4}u^{2}}{{d_{0}}^{2}},\\ \sigma_{3}=-{\varsigma \varsigma }_{0}\frac{\lambda_{1}^{4}u^{2}}{{d_{0}}^{2}}. \end{array}$$

#### 4.3.2. Nine-Parameter Mooney-Rivlin Model

From (9), the strain energy equation in the nine-parameter Mooney-Rivlin model is

(57) $$\begin{array}{c} W=c_{10}\left(I_{1}-3\right)+c_{01}\left(I_{2}-3\right)+c_{20}{\left(I_{1}-3\right)}^{2}+c_{11}\left(I_{1}-3\right)\left(I_{2}-3\right)+c_{02}{\left(I_{2}-3\right)}^{2}+c_{30}{\left(I_{1}-3\right)}^{3}+c_{21}{\left(I_{1}-3\right)}^{2}\left(I_{2}-3\right)+c_{12}\left(I_{1}-3\right){\left(I_{2}-3\right)}^{2}+c_{03}{\left(I_{2}-3\right)}^{3}. \end{array}$$

For (57), the partial differentials of the strain energy function *W* for *I*_1_ and *I*_2_ are given by

(58) $$\begin{array}{c} \frac{\partial W}{\partial I_{1}}=c_{10}+2c_{20}\left(I_{1}-3\right)+c_{11}\left(I_{2}-3\right)+3c_{30}{\left(I_{1}-3\right)}^{2}+2c_{21}\left(I_{1}-3\right)\left(I_{2}-3\right)+c_{12}{\left(I_{2}-3\right)}^{2}=M,\\ \frac{\partial W}{\partial I_{2}}=c_{01}+2c_{02}\left(I_{2}-3\right)+c_{11}\left(I_{1}-3\right)+3c_{03}{\left(I_{2}-3\right)}^{2}+2c_{12}\left(I_{1}-3\right)\left(I_{2}-3\right)+c_{21}{\left(I_{1}-3\right)}^{2}=N. \end{array}$$

Because the materials uniformly stretched in the *X* and *Y* directions,

(59) $$\begin{array}{c} \sigma_{1}=\sigma_{2}. \end{array}$$

From (41),

(60) $$\begin{array}{c} \sigma_{3}=-{\varsigma \varsigma }_{0}V^{2}=-{\varsigma \varsigma }_{0}\frac{u^{2}}{d^{2}}=-{\varsigma \varsigma }_{0}\frac{u^{2}}{{\left(\lambda_{3}d_{0}\right)}^{2}}. \end{array}$$

Substituting (58), (59), and (60) into (44) and (46),

(61) $$\begin{array}{c} \sigma_{1}=\sigma_{2}=2\left(\lambda_{1}^{2}M-\frac{1}{\lambda_{1}^{2}}N\right)-p, \end{array}$$

(62) $$\begin{array}{c} \sigma_{3}=2\left(\frac{1}{\lambda_{1}^{4}}M-\lambda_{1}^{4}N\right)-p=-{\varsigma \varsigma }_{0}\frac{u^{2}}{{\left(\lambda_{3}d_{0}\right)}^{2}}=-{\varsigma \varsigma }_{0}\frac{\lambda_{1}^{4}u^{2}}{{d_{0}}^{2}}. \end{array}$$

From (61) and (62),

(63) $$\begin{array}{c} p=2\left(\frac{1}{\lambda_{1}^{4}}M-\lambda_{1}^{4}N\right)+{\varsigma \varsigma }_{0}\frac{\lambda_{1}^{4}u^{2}}{{d_{0}}^{2}}. \end{array}$$

Therefore,

(64) $$\begin{array}{c} \sigma_{1}=\sigma_{2}=2\left(\lambda_{1}^{2}M-\frac{1}{\lambda_{1}^{2}}N\right)-2\left(\frac{1}{\lambda_{1}^{4}}M-\lambda_{1}^{4}N\right)-{\varsigma \varsigma }_{0}\frac{\lambda_{1}^{4}u^{2}}{{d_{0}}^{2}},\\ \sigma_{3}=-{\varsigma \varsigma }_{0}\frac{\lambda_{1}^{4}u^{2}}{{d_{0}}^{2}}. \end{array}$$

## 5. Experimental Analysis

### 5.1. Production of Tensile Test Sample

**Figure 4:**
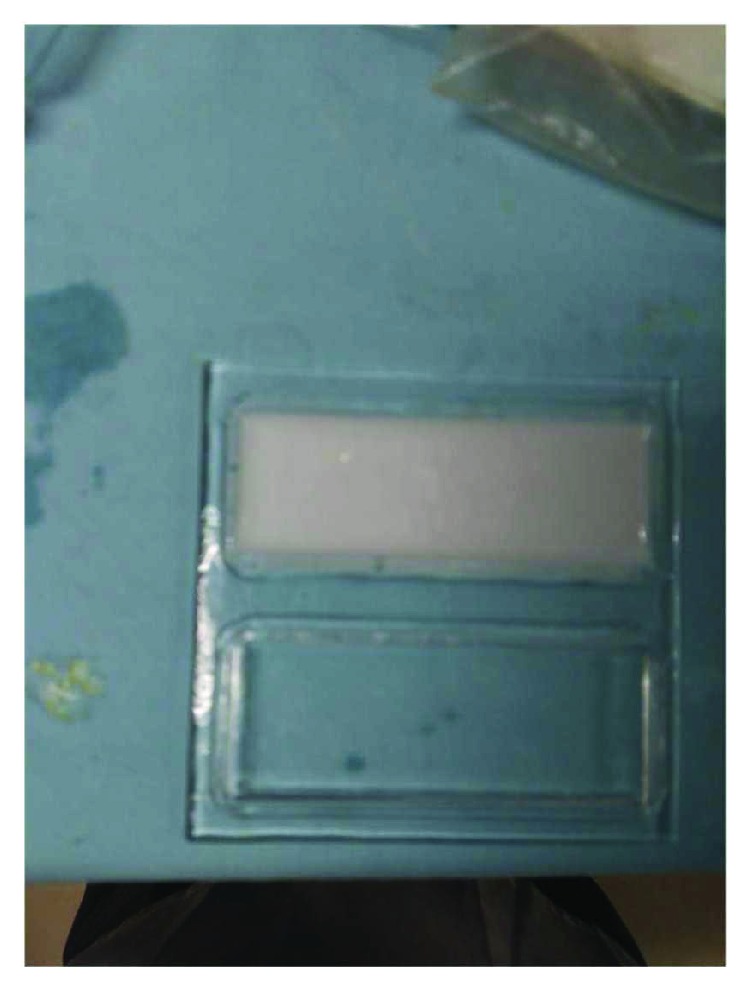
The gel after polymerization under a UV lamp.

**Figure 5:**
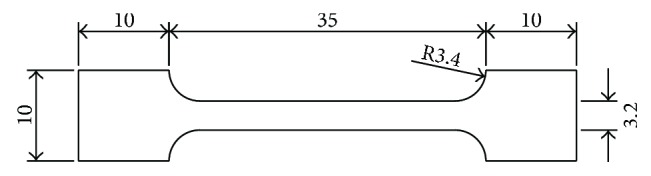
Test sample size (in mm).

**Figure 6:**
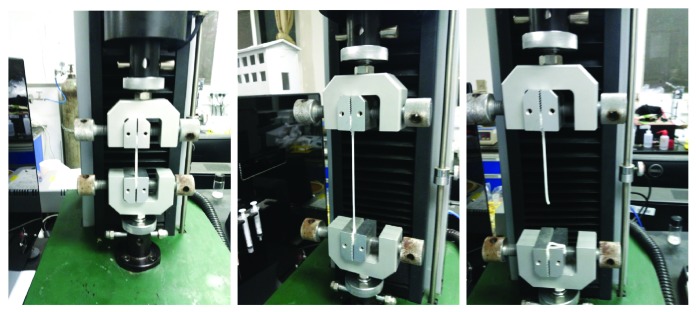
Uniaxial tensile test.

The prepared solution was poured into a mold and polymerized into a gel under the irradiation of a UV lamp. The gel is white in color and is filled in the transparent glass mold as shown in Figure 4. Then, the sample was cut to the size shown in Figure 5, with a thickness of 3.4 mm. As shown in Figure 6, the experimental instrument was a UTM2502 electronic universal testing machine. The mechanical sensor on the testing machine can achieve a precision of 0.1 mN, the displacement sensor has a precision of 0.001 mm, and the stretching rate is 500 mm/min.

### 5.2. Experimental Results

As seen from Figure 6, the ILG becomes longer and thinner as the load increases, which is consistent with the assumption that the material is incompressible.

**Figure 7:**
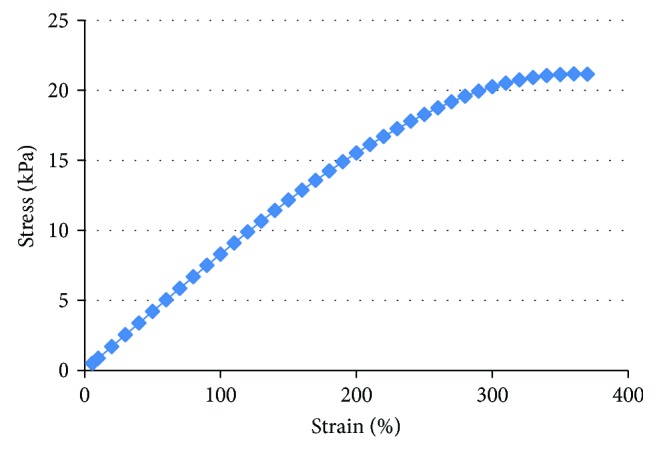
Stress-strain curve of the ionic liquid gel polymer.

The tensile stress-strain curve of the ILG is shown in Figure 7, and the average tensile strength (Young's modulus) of the material obtained from the tensile stress-strain curve is 7.6 kPa.

In the ionogel, BMIMBF_4_ exhibits a high level of hyperelastic toughness when the tensile deformation reaches 360%. The tensile test showed that the tensile properties of the gel increased with an increase in ZrO_2_ content. The increase in ZrO_2_ content should generate more cross-linking sites and higher conversion rates (shown in Figure 2), which contributes to the overall mechanical properties. As the content of ZrO_2_ increases, the tensile strength of the ILG increases, while the elongation rate decreases. Considering the above data, the optimum amount of ZrO_2_ to provide a large tensile strain and tensile strength is 3 wt%.

### 5.3. Stress Calculation

The relationship between the real stress *σ*_*i*_ and engineering stress *σ*_E_ is

(65) $$\begin{array}{c} \sigma_{i}=\sigma_{E}\lambda , \end{array}$$

where *σ*_*i*_ is the true stress and *σ*_E_ is the engineering stress. The calculated stress given in Table 1 is the engineering stress.

We next tested whether the above-derived engineering stress expression for the ILG is accurate and whether it can be used for the design of an ILG actuator or sensor. To verify the accuracy and practicability of the induced stress expression of the ILG, the deformation rate at each point in the uniaxial tensile test was substituted into the engineering stress expression to calculate the engineering stress. A comparison of the calculated uniaxial tensile stress with the experimental data is given in Table 1.

For the five-parameter Mooney-Rivlin model, the parameters *c*_10_, *c*_01_, *c*_20_, *c*_11_, and *c*_02_ are as follows.

(66) $$\begin{array}{c} c_{10}=2.3582,\\ c_{01}=-0.87482,\\ c_{20}=-0.066187,\\ c_{11}=0.19663,\\ c_{02}=0.21939. \end{array}$$

For the nine-parameter Mooney-Rivlin model, the parameters *c*_10_, *c*_01_, *c*_20_, *c*_11_, *c*_02_, *c*_30_, *c*_21_, *c*_12_, and *c*_03_ are as follows.

(67) $$\begin{array}{c} c_{10}=0.55361,\\ c_{01}=1.0009,\\ c_{20}=130.08,\\ c_{11}=-270.06,\\ c_{02}=142.91,\\ c_{30}=0.014438,\\ c_{21}=-0.17711,\\ c_{12}=-31.71,\\ c_{03}=17.028. \end{array}$$

As seen from Table 1 in the comparisons of the calculated tensile stress with the experimental data, the relative error of the five-parameter Mooney-Rivlin model is less than 0.51% and the relative error of the nine-parameter Mooney-Rivlin model is no more than 8.87%.

**Figure 8:**
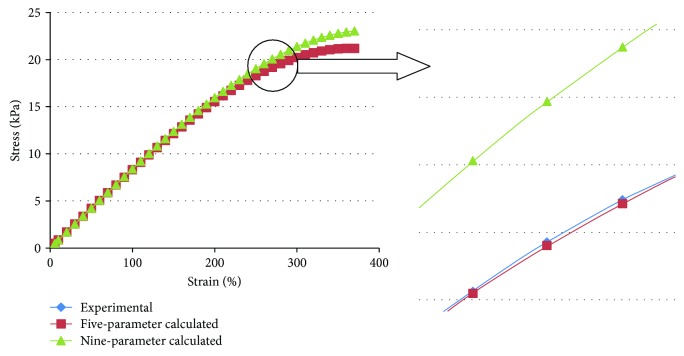
Comparison of the calculated tensile stress with the experimental data from the uniaxial tensile test.

Figure 8 shows that the stress-strain curve calculated by the five-parameter model almost coincides with the experimental curve. The first half of the stress-strain curve calculated by the nine-parameter model almost coincides with the experimental curve, while in the second half, the error between the stress-strain curve calculated by the nine-parameter model and the experimental curve becomes increasingly larger, but the error remains small.

The above analyses indicate that the simulated values are consistent with the experimental values, making our derivation a feasible and credible stress formula for the calculation of the ILG properties.

Due to our current laboratory conditions, only the uniaxial tensile test was performed. The next step is to improve the laboratory conditions in order to carry out the plane uniform tensile test and the Maxwell stress experiments. Further studies of the five-parameter and nine-parameter Mooney-Rivlin models will be carried out.

## 6. Conclusions

In this paper, an ILG is modeled by the hyperelastic nonlinear finite element model. The simulation results show that the Mooney-Rivlin model can well adapt to the constitutive relation of the material [30, 31]. The main advantage of the ILG is that the stress-strain curve can be obtained by the performance parameters of the material in a relatively short time, which provides a theoretical basis for the optimal design of a soft robot.

The simulation results show that the average error between the calculated data and the experimental data is small, and the model has a good correlation with the experimental data. The model requires the input of the ILG material parameters. A standard uniaxial stretching method is used to obtain the desired ILG material parameters.

In the future, we will seek a generalized algorithm for identifying the ILG mechanical properties. Notably, all the results of this study show that there is a good correlation between the 3D theoretical assumptions and the experimental conditions, which proves that our method can be used to optimize the design of a soft robot. This work opens a new path to study the performance of ILG soft actuators, which will be the direction of future work.

## Figures and Tables

**Table 1:** Comparison of the calculated tensile stress with the experimental data from the uniaxial tensile test.

Measurement data		Experimental results		Five-parameter Mooney-Rivlin model		Nine-parameter Mooney-Rivlin model	
Force (mN)	Deformation (mm)	Strain (%)	Stress (kPa)	Calculated stress (*σ*_E_) (kPa)	Error (%)	Calculated stress (*σ*_E_) (kPa)	Error (%)
4.97	2.0	5.7	0.50	0.50	0.46	0.50	0.00
8.21	3.5	10	0.86	0.86	0.51	0.86	0.07
14.88	7.0	20	1.70	1.70	0.26	1.70	0.03
20.52	10.5	30	2.54	2.54	0.06	2.55	0.26
25.35	14.0	40	3.38	3.37	0.24	3.39	0.29
29.47	17.5	50	4.21	4.21	0.13	4.23	0.44
33.06	21.0	60	5.04	5.04	0.09	5.07	0.49
36.22	24.5	70	5.86	5.86	0.05	5.90	0.68
38.94	28.0	80	6.68	6.69	0.07	6.73	0.8
41.48	31.5	90	7.50	7.50	0.01	7.57	0.87
43.58	35.0	100	8.30	8.30	0.05	8.39	1.10
45.50	38.5	110	9.10	9.10	0.01	9.21	1.21
47.13	42.0	120	9.88	9.88	0.02	10.02	1.41
48.67	45.5	130	10.65	10.65	0.01	10.82	1.57
49.98	49.0	140	11.41	11.41	0.04	11.60	1.67
51.03	52.5	150	12.15	12.14	0.06	12.37	1.80
51.95	56.0	160	12.86	12.86	0.01	13.12	2.03
52.75	59.5	170	13.56	13.56	0.01	13.86	2.19
53.40	63.0	180	14.24	14.24	0.0	14.58	2.35
53.90	66.5	190	14.89	14.90	0.05	15.27	2.58
54.32	70.0	200	15.52	15.53	0.06	15.95	2.79
54.65	73.5	210	16.12	16.14	0.11	16.61	3.05
54.78	77.0	220	16.70	16.72	0.10	17.25	3.29
54.86	80.5	230	17.25	17.27	0.11	17.86	3.55
54.94	84.0	240	17.78	17.79	0.07	18.45	3.78
54.84	87.5	250	18.28	18.28	0.01	19.02	4.02
54.72	91.0	260	18.74	18.74	0.00	19.55	4.32
54.47	94.5	270	19.18	19.16	0.08	20.05	4.56
54.01	98.0	280	19.57	19.55	0.09	20.53	4.89
53.61	101.5	290	19.93	19.90	0.14	20.97	5.19
53.23	105.0	300	20.24	20.21	0.13	21.37	5.57
52.48	108.5	310	20.50	20.49	0.07	21.73	6.00
51.83	112.0	320	20.73	20.71	0.08	22.05	6.38
51.02	115.5	330	20.91	20.90	0.05	22.34	6.81
50.29	119.0	340	21.04	21.04	0.01	22.57	7.29
49.21	122.5	350	21.12	21.13	0.06	22.77	7.80
48.25	126.0	360	21.16	21.18	0.08	22.92	8.31
47.17	129.5	370	21.15	21.17	0.10	23.03	8.87

## References

[1] Fukushima T., Asaka K., Kosaka A., Aida T. (2005). Fully plastic actuator through layer-by-layer casting with ionic-liquid-based bucky gel.

[2] Kruusamäe K., Mukai K., Sugino T., Asaka K. (2014). Mechanical behaviour of bending bucky-gel actuators and its representation.

[3] He Q., Yu M., Yang X., Kim K. J., Dai Z. (2015). An ionic electro-active actuator made with graphene film electrode, chitosan and ionic liquid.

[4] Pelrine R., Kornbluh R., Pei Q., Joseph J. (2000). High-speed electrically actuated elastomers with strain greater than 100%.

[5] Hammock M. L., Chortos A., Tee B. C.-K., Tok J. B.-H., Bao Z. (2013). 25th anniversary article: the evolution of electronic skin (E-skin): a brief history, design considerations, and recent progress.

[6] Feinberg A. W., Feigel A., Shevkoplyas S. S., Sheehy S., Whitesides G. M., Parker K. K. (2007). Muscular thin films for building actuators and powering devices.

[7] Liu G., Wang A., Wang X., Liu P. (2016). A review of artificial lateral line in sensor fabrication and bionic applications for robot fish.

[8] Buchtová N., Guyomard-Lack A., Le Bideau J. (2014). Biopolymer based nanocomposite ionogels: high performance, sustainable and solid electrolytes.

[9] Le Bideau J., Viau L., Vioux A. (2011). Ionogels, ionic liquid based hybrid materials.

[10] Zhang W., Chen L., Zhang J., Huang Z. (2017). Design and optimization of carbon nanotube/polymer actuator by using finite element analysis.

[11] Zhang M., Xu D., Yan X. (2012). Self-healing supramolecular gels formed by crown ether based host–guest interactions.

[12] Zhang D., Yang J., Bao S., Wu Q., Wang Q. (2013). Semiconductor nanoparticle-based hydrogels prepared via self-initiated polymerization under sunlight, even visible light.

[13] Leski A., Baraniecki R., Malachowski J. Numerical simulation to study the influence of the thickness of canopy at a bird strike.

[14] He F., Hua L., Gao L. J. (2015). A computational model for biomechanical effects of arterial compliance mismatch.

[15] Lee S., Park H. C., Kim K. J. (2005). Equivalent modeling for ionic polymer-metal composite actuators based on beam theories.

[16] Wang Z., He B., Wang Q., Yin Y. (2016). Electromechanical bending behavior study of soft photocurable ionogel actuator using a new finite element method.

[17] He B., Zhang C.-H., Ding A. (2017). Finite element analysis of ionic liquid gel soft actuator.

[18] Alexandrov S., Miszuris W. (2016). Heat generation in plane strain compression of a thin rigid plastic layer.

[19] Sonato M., Piccolroaz A., Miszuris W., Mishuris G. (2015). General transmission conditions for thin elasto-plastic pressure-dependent interphase between dissimilar materials.

[20] Liu X., He B., Wang Z., Tang H., Su T., Wang Q. (2015). Tough nanocomposite ionogel-based actuator exhibits robust performance.

[21] Li M., Yang Y., Guo L., Chen D., Sun H., Tong J. (2015). Design and analysis of bionic cutting blades using finite element method.

[22] Liu Y., Liu L., Sun S., Leng J. (2010). Electromechanical stability of a Mooney–Rivlin-type dielectric elastomer with nonlinear variable permittivity.

[23] Liu L., Liu Y., Yu K., Leng J. (2014). Thermoelectromechanical stability of dielectric elastomers undergoing temperature variation.

[24] Sangpradit K., Liu H., Dasgupta P., Althoefer K., Seneviratne L. D. (2011). Finite-element modeling of soft tissue rolling indentation.

[25] Prados-Privado M., Bea J. A., Rojo R., Gehrke S. A., Calvo-Guirado J. L., Prados-Frutos J. C. (2017). A new model to study fatigue in dental implants based on probabilistic finite elements and cumulative damage model.

[26] Liu Y., Liu L., Zhang Z., Jiao Y., Sun S., Leng J. (2010). Analysis and manufacture of an energy harvester based on a Mooney-Rivlin–type dielectric elastomer.

[27] Yeoh O. H. (1993). Some forms of the strain energy function for rubber.

[28] Facchinetti M., Miszuris W. (2016). Analysis of the maximum friction condition for green body forming in an ANSYS environment.

[29] Hu D., Song B., Wang D., Chen Z. (2016). Experiment and numerical simulation of a full-scale helicopter composite cockpit structure subject to a bird strike.

[30] Sun W., Chaikof E. L., Levenston M. E. (2008). Numerical approximation of tangent moduli for finite element implementations of nonlinear hyperelastic material models.

[31] Ihueze C., Mgbemena C. (2014). Modeling hyperelastic behavior of natural rubber/organomodified kaolin composites oleochemically derived from tea seed oils (*Camellia sinensis*) for automobile tire side walls application.

